# Reception of the Wounded at St. Thomas's Hospital

**Published:** 1914-09-12

**Authors:** 


### Reception of the Wounded at St. Thomas's Hospital.

On Thursday evening last week, September 3, the authorities at St. Thomas's Hospital were notified that a train-load of wounded was arriving at Waterloo from Southampton, and it was expected that the train would leave about 6 p.m. There was evidently some delay in crossing, as it was 9.30 p.m. before the train got away from Southampton, and nearly midnight before it arrived at Waterloo.

Colonel Skinner, chief of the London Medical Department, was in charge of the arrangements at the arrival platform, and he was accompanied by Major Hawkins, the senior physician, and Captain C. A. Ballance, a surgeon of the hospital. The St. John Ambulance furnished stretcher-bearers. There was a fleet of motor-cars and horse ambulances under the organisation of the Red Cross Society, the details of which are under the charge of Mr. and Mrs. Clinton Dent.

#### "All Quiet by 2 a.m."

A huge crowd at the station had to be kept back both by the military and the police, but the removal of the wounded and sick from the train to the ambulances was very expeditiously managed, and they were quickly taken down to the hospital. An efficient staff of porters, aided by medical students, many of whom are members of the R.A.M.C. "T." and trained as stretcher-bearers, were in attendance. Within a quarter of an hour all those who were able to walk or who only needed a wheel-chair were taken to the three wards allotted to them. There was a slight interval before the horse ambulances arrived, but all were in their wards well within the hour from the time of the arrival of the train at Waterloo. In each ward the sisters were called and took charge under the supervision of the matron, Miss Lloyd-Still, and thoroughly efficient arrangements had been made for providing hot baths for every man able to take a bath, and a hot meal was provided for each. Many looked very travel-worn and hungry. The physicians and surgeons of the hospital who are on the staff of No. 2 London General Hospital, which is established at St. Mark's College, Chelsea, went over all the cases with the resident assistant physician and the resident assistant surgeon, and by 2 A.M. the wards were quiet and all were at rest.

### Three Wards Occupied.

Three of the wards on the main first floor have been taken for the accommodation of these patients—an average of forty in each. One commander of the Royal Navy is amongst those brought to the hospital. One is an ophthalmic case, and five others are being treated in the isolation ward owing to septic conditions. Colonel Callender, the chief medical officer in charge of No. 2, visited the hospital on Friday last week and inspected the arrangements. The men are regarded as on active service and under military discipline, the general rules of the hospital being fully observed.

### The Nature of the Injuries.

Though there are not very severe wounds amongst the men under treatment, all bear evidence of the hardships which they have endured. Men were found who had not had their boots or puttees off since they landed in France. Many were suffering from strains and sore feet, the results of marching, while there were remarkably few actual bullet wounds, though some were injured by shrapnel. The largest number of these wounded came from the Royal Scots, the 4th Middlesex, the East Surrey, and the 2nd battalion of the Essex Regiment. The men all seemed to have a very poor opinion of the rifle shooting of the Germans, but speak of the danger of the artillery fire. Most of the cavalry who are in the wards were injured through their horses rolling on them when struck either by bullets or by shrapnel. Several had been in bayonet charges, and spoke of the great evidence of superiority of the English troops with the bayonet, the Germans evidently shirking this form of attack on all occasions. Colonel Skinner inspected the wards on Saturday morning, and later in the afternoon Lord Kitchener (as we also note elsewhere) visited the men and cheered them on by his advice and an appeal to get well as quickly as possible and get back to the front. No words were needed for this, all being anxious to get in again, for, as they say, "We have them all licked."

### Visits to the Soldiers.

All who had friends were visited on Sunday at the usual hours, but many whose friends live at a distance—Scotland, the North of England, the West of England, etc.—are anxious for visits from their relations, and any funds sent to help these relatives in coming to visit the soldiers before they are obliged to return to duty will be very gladly received by the secretary of the hospital and devoted to the purpose for which they are sent. This work is being efficiently managed by the Northcote Trust, who are trained organisers of this particular work.

His Majesty the King, accompanied by the Queen, gave notice at 10.30 on Monday morning that they would visit the hospital at 12 o'clock. They arrived punctually, attended by Sir Charles Cust. He was met at the entrance of the hospital by the secretary, Mr. G. Q. Roberts, the matron, Miss Lloyd-Still, the senior physician, Major Hawkins, and the senior surgeon, Lieut.-Colonel G. H. Makins. In each ward the medical officers in charge of the cases—Captain Mackenzie, Captain Turney, Captain Ballance, and Captain Robinson—were in attendance and answered any questions from his Majesty as to the nature of the cases. Their Majesties cheered all by their very kind sympathy and inquiries. Cheers were given for their Majesties as they entered and as they left the wards. After a visit of one and a half hours they left the hospital expressing the warmest approval of the arrangements for the comfort of the sick and wounded. The King's visit is also referred to in our Notes this week.

On Tuesday afternoon their Royal Highnesses Princess Christian and Princess Henry of Battenburg visited the hospital accompanied by the Princesse de Villevorde and her two daughters. Every patient was visited, the Belgian Princess being particularly interested in, and most sympathetic with, the wounded whom she saw.

### How the Civil Population are Cared for.

The hospital is fortunately able to carry on its full work of attending to the civil population without any diminution, owing to the fact that Lady Henry Somerset, at Duxhurst, has placed her hospital at the disposal of the authorities, and cases being treated in the wards, which no longer need special treatment at the hospital, can be sent to this home much sooner than usual, where they receive efficient and all necessary treatment. The kindness of Lady Henry Somerset in thus coming to their aid is very much appreciated by the authorities of the hospital, who are thus able to render valuable service to their country in this terrible time of trial and not to diminish in any degree whatever the usual work of the hospital.

